# Estimating the hospitalization burden associated with influenza and respiratory syncytial virus in New York City, 2003–2011

**DOI:** 10.1111/irv.12325

**Published:** 2015-08-04

**Authors:** Edward Goldstein, Sharon K Greene, Donald R Olson, William P Hanage, Marc Lipsitch

**Affiliations:** a Department of Epidemiology, Center for Communicable Disease Dynamics, Harvard School of Public HealthBoston, MA, USA; b Bureau of Communicable Disease, New York City Department of Health and Mental HygieneQueens, NY, USA; c Bureau of Epidemiology Services, New York City Department of Health and Mental HygieneQueens, NY, USA; d Department of Immunology and Infectious Disease, Harvard School of Public HealthBoston, MA, USA

**Keywords:** Hospitalization, influenza, New York City, respiratory syncytial virus

## Abstract

**Background:**

Hospitalization burden associated with influenza and respiratory syncytial virus (RSV) is uncertain due to ambiguity in the inference methodologies employed for its estimation.

**Objectives:**

Utilization of a new method to quantitate the above burden.

**Methods:**

Weekly hospitalization rates for several principal diagnoses from 2003 to 2011 in New York City by age group were regressed linearly against incidence proxies for the major influenza subtypes and RSV adjusting for temporal trends and seasonal baselines.

**Results:**

Average annual rates of influenza-associated respiratory hospitalizations per 100 000 were estimated to be 129 [95% CI (79, 179)] for age <1, 36·3 (21·6, 51·4) for ages 1–4, 10·6 (7·5, 13·7) for ages 5–17, 25·6 (21·3, 29·8) for ages 18–49, 65·5 (54·0, 76·9) for ages 50–64, 125 (105, 147) for ages 65–74, and 288 (244, 331) for ages ≥75. Additionally, influenza had a significant contribution to hospitalization rates with a principal diagnosis of septicemia for ages 5–17 [0·76 (0·1, 1·4)], 18–49 [1·02 (0·3, 1·7)], 50–64 [4·0 (1·7, 6·3)], 65–74 [8·8 (2·2, 15·6)], and ≥75 [38·7 (25·7, 52·9)]. RSV had a significant contribution to the rates of respiratory hospitalizations for age <1 [1900 (1740, 2060)], ages 1–4 [117 (70, 167)], and ≥75 [175 (44, 312)] [including chronic lower respiratory disease, 90 (43, 140)] as well as pneumonia & influenza hospitalizations for ages 18–49 [6·2 (1·1, 11·3)] and circulatory hospitalizations for ages ≥75 [199 (13, 375)].

**Conclusions:**

The high burden of RSV hospitalizations among young children and seniors age ≥75 suggests the need for additional control measures such as vaccination to mitigate the impact of annual RSV epidemics. Our estimates for influenza-associated hospitalizations provide further evidence of the burden of morbidity associated with influenza, supporting current guidelines regarding influenza vaccination and antiviral treatment.

## Introduction

Estimating the burden of severe outcomes associated with influenza and respiratory syncytial virus in various population groups is important to inform mitigation efforts, including potential development of an RSV vaccine.^1^–^4^ With most hospitalizations associated with influenza and RSV infections missing a mention of those pathogens in the diagnoses, substantial uncertainty still exists about the magnitude of this burden and the statistical method of estimating it.^5^–^9^ The aim of our work was to both address the statistical issues related to the estimation of the hospitalization burden associated with influenza and RSV and exhibit a detailed picture of this burden by considering a number of principal hospitalization diagnoses that have a potential contribution from influenza and RSV.

While much of the related recent work on estimating the severe outcome burden associated with influenza and RSV relies on regression analysis,^10^–^15^ several aspects of such methodologies are questionable. One is that the commonly employed model assumes a nonlinear relation between exposure (influenza incidence rates) and outcome (influenza-attributable hospitalization)^6^,^16^ while the rates of severe outcomes associated with influenza are expected to be proportional to influenza incidence rates, and the implications of modeling the relation between incidence and severe outcomes in a nonlinear fashion are uncertain.^13^ Many estimates also use a sinusoidal model to account for baseline rates of outcomes not associated with respiratory viruses.^6^,^8^,^17^ This assumption might be more problematic for RSV compared with influenza due to the high year-to-year periodicity of RSV circulation (particularly compared with influenza,^7^ which could confound the estimation of RSV-attributable outcomes if the true baseline varies in a fashion correlated with RSV.

The approach proposed in ref.^13^ was designed to overcome these problems, employing a linear relation between the rates of influenza incidence and severe outcomes and adopting a flexible model for the baseline rates of severe outcomes not associated with influenza. Here, we apply a similar approach to the New York City data between 2003 and 2011, adding an appropriate RSV incidence proxy to the inference model in ref.^13^ This inference method is applied to data on respiratory hospitalizations [including hospitalization with principal diagnoses of pneumonia and influenza (P&I) and chronic lower respiratory disease (CLRD)] as well as for hospitalization with a number of other principal diagnoses (including circulatory causes) in different age groups in New York City between 2003 and 2011 to estimate the burden for the above categories of hospitalization associated with influenza and RSV.

## Methods

Article 28 of New York State law requires general hospitals to submit inpatient hospitalization data to the New York State Statewide Planning and Research Cooperative System.^18^ We used weekly counts of all hospitalizations among New York City (NYC) residents who were discharged from NYC hospitals with various principal diagnosis codes, reflecting the condition chiefly responsible for the patient’s admission.^19^ Only the principal/primary diagnosis was considered, not other diagnoses that coexisted at the time of admission or developed subsequently. Following our approach in ref.,^13^ the principal diagnosis categories we considered were respiratory causes, P&I, CLRD, all circulatory causes, diabetes, renal disease, Alzheimer’s disease, and septicemia (see Supporting information). Data were stratified into seven age groups: ages <1, 1–4, 5–17, 18–49, 50–64, 65–74, and ≥75 years. For respiratory hospitalizations among ages <1, 1–4, and 5–17, hospitalizations with a principal diagnosis of asthma or croup were removed from the data due to the high aperiodicity in those hospitalization categories as suggested by ref.,^20^ resulting in improved model fits. The removal of asthma hospitalizations left very low weekly counts for the pediatric CLRD hospitalizations, so the analysis of influenza- and RSV-associated CLRD hospitalizations was restricted to the adult age groups.

The period analyzed was between calendar week 36 of 2003 and calendar week 34, 2011, with calendar week 27, 2008 discarded to ensure a 52-week periodicity each season. An influenza season was defined as calendar week 40 through week 20 of the next year, except for the spring/summer of 2009 (weeks 16–34, 2009); separate estimates were calculated for this period corresponding to the first wave of the A/H1N1 influenza pandemic. We defined the weekly incidence proxies for the major influenza (sub) types (A/H1N1, A/H3N2 and B) and RSV as follows: the weekly incidence proxy for an influenza subtype was the percent of emergency department (ED) visits in NYC classified as influenza-like illness multiplied by the percent of respiratory specimens which were positive for a given subtype; the weekly incidence proxy for RSV was the rate of hospitalizations coded for RSV bronchiolitis for age <1. To adjust for potential temporal inconsistency between the influenza proxies and the rates of hospitalizations associated with the corresponding influenza subtypes (a temporally varying ratio of those two quantities for certain combinations of an influenza subtype/age group), we have essentially split those proxies into several time periods (see Supporting information for full details). We have employed the following inference method adopted from ref.^13^

### Main inference method

For each principal diagnosis category of hospitalization and age group, weekly hospital admission rates *H*(*t*) (per 100 000 individuals in that age group^21^) were regressed linearly against the weekly incidence proxies Flu_*i*_(*t*) and RSV(*t*) for the major influenza subtypes and RSV (see Supporting information), a temporal trend (modeled by a low degree polynomial in time) and a seasonal baseline (modeled by periodic cubic splines with a period of 1 year): 

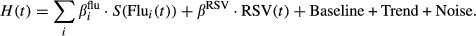
1

Here *S*() is a forward shift between 0 and 1 weeks reflecting the lag between influenza infection and hospitalization as explained in the preliminary step below. While the noise in eq. 1 is autocorrelated, ordinary least squares (OLS) were used in eq. 1 to produce the model’s estimates due to the fact that those are unbiased regardless of the autocorrelation structure for the noise.^13^ A bootstrap procedure with the noise modeled as a first-order autoregressive AR(1) process was employed to produce the confidence bounds for the OLS estimates.^13^

### Preliminary step

For each number 0 ≤ *a* ≤ 1, the forward shift operator *S*_*a*_ is defined as follows: 

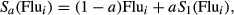
2where *S*_*i*_ is the forward shift of the weekly influenza incidence proxy by 1 week. The number 0 ≤ *a* ≤ 1 is chosen so that the *R*^2^ for the corresponding OLS fit given by eq. 1 is minimized. This number is then used for the definition of the forward shift operator *S* in eq. 1.

### Alternative inference methods

In the Supporting information, we consider a number of alternative inference models, examining the sensitivity of the Main Inference Method with regard to the choice of the incidence proxies, the model for the baseline, the issue of hospitalization counts versus hospitalization rates, etc.

## Results

Here, we present the results for the Main Inference Method above.

### Respiratory causes

Here, we present the estimates for influenza’s and RSV’s contribution to the rates of all respiratory, P&I, and CLRD hospitalizations, with model fits in those categories shown in the Supporting information.

#### All respiratory hospitalizations

Table1 presents the annual (including spring/summer 2009, corresponding to the first wave of the A/H1N1 pandemic) and average annual estimates (the total divided by 8) and 95% confidence intervals for influenza- and RSV-associated respiratory hospitalization rates.

**Table 1:** Estimated annual and average annual rates per 100 000 of influenza (Flu)- and RSV-associated respiratory hospitalizations in different age groups, 2003–2011

Season/Age group (years)		<1	1–4	5–17	18–49	50–64	65–74	≥75
2003–2004	Flu	435·2 (376, 499)	84·5 (65·5, 104)	10·5 (6·4, 14·7)	24·2 (18·5, 29·8)	76·6 (60·8, 92·1)	155·6 (128, 183)	337·4 (283, 398)
	RSV	2126·4 (1958, 2301)	127·5 (76·1, 183)	1·6 (−10·5, 13·8)	13·3 (−2·3, 28·6)	29·8 (−11, 70)	16·7 (−63·4, 92·4)	191·1 (47·6, 341)
2004–2005	Flu	124·7 (36·1, 218)	−0·5 (−28·7, 27·9)	4·6 (−1·5, 10·6)	32·6 (24·4, 40·9)	117·4 (94·7, 140)	269·6 (228, 311)	713·5 (632, 799)
	RSV	1726·1 (1589, 1868)	103·5 (61·8, 148)	1·3 (−8·5, 11·2)	10·8 (−1·9, 23·2)	24·2 (−8·9, 56·8)	13·5 (−51·5, 75)	155·1 (38·6, 277)
2005–2006	Flu	28·7 (−34·5, 91·7)	13·9 (−5·2, 34·3)	5·7 (1·6, 10)	18·5 (12·9, 24·2)	46·3 (30·7, 61·6)	111·1 (82·5, 138)	257·3 (197, 314)
	RSV	1997·8 (1840, 2162)	119·8 (71·5, 172)	1·5 (−9·8, 13)	12·5 (−2·2, 26·8)	28·0 (−10·3, 65·7)	15·7 (−59·6, 86·8)	179·5 (44·7, 321)
2006–2007	Flu	50·8 (−5·7, 105)	5·5 (−11·1, 22·8)	4·4 (1·0, 7·9)	21·2 (16·2, 26)	42·6 (29, 55·8)	83·3 (59·6, 106)	193·5 (143, 240)
	RSV	2092·2 (1927, 2264)	125·5 (74·9, 180)	1·6 (−10·3, 13·6)	13·1 (−2·3, 28·1)	29·4 (−10·8, 68·8)	16·4 (−62·4, 90·9)	188·0 (46·8, 336)
2007–2008	Flu	63·6 (−28·0, 152)	7·4 (−19·4, 35·7)	7·6 (1·8, 13·1)	29·9 (22·1, 37·7)	67·1 (45·4, 88·6)	135·1 (96·5, 173)	351·2 (271, 428)
	RSV	1606·6 (1479, 1739)	96·4 (57·5, 138)	1·2 (−7·9, 10·4)	10·0 (−1·8, 21·6)	22·5 (−8·3, 52·9)	12·6 (−47·9, 69·8)	144·4 (36, 258)
2008–2009	Flu	57·8 (−11, 126)	−0·6 (−22·6, 20·7)	3·5 (−0·8, 7·9)	20·7 (14·6, 26·6)	37·4 (20·9, 54·2)	60·6 (30·8, 91·5)	150·1 (85·8, 210)
	RSV	1796·8 (1655, 1945)	107·8 (64·3, 154)	1·3 (−8·8, 11·7)	11·2 (−2, 24·1)	25·2 (−9·3, 59·1)	14·1 (−53·6, 78·1)	161·5 (40·2, 288)
Spring/Summer 2009	Flu	142·1 (90·9, 193)	55·9 (39·2, 72·3)	32·8 (29·5, 36·3)	27·5 (23, 32·1)	48·2 (35·1, 60·8)	47·3 (23·7, 70·8)	23·0 (−23, 72·4)
	RSV	63·8 (60·1, 70·6)	3·91 (2·3, 5·6)	0·1 (−0·3, 0·4)	0·4 (−0·1, 0·9)	0·9 (−0·3, 2·1)	0·5 (−1·9, 2·8)	5·9 (1·5, 10·5)
2009–2010	Flu	61·3 (−97·7, 220)	100·5 (66·3, 134)	6·8 (−0·1, 14·1)	13·8 (4·4, 23·4)	12·5 (−13·1, 38·2)	29·2 (−16·3, 77·5)	−56·6 (−152, 41·8)
	RSV	2057·4 (1808, 2320)	136·5 (81·5, 195)	1·7 (−11·2, 14·8)	14·2 (−2·5, 30·6)	31·9 (−11·8, 74·9)	17·8 (−67·9, 98·9)	204·6 (51, 365)
2010–2011	Flu	68·1 (−61·9, 199)	24·2 (−6·2, 55·8)	9·1 (2·9, 15·9)	16·2 (7·3, 25)	75·6 (51·5, 99·3)	114·5 (69·3, 159)	337·1 (248, 428)
	RSV	1699·09 (1493, 1916)	112·8 (67·3, 161)	1·4 (−9·3, 12·2)	11·7 (−2·1, 25·2)	26·4 (−9·7, 61·9)	14·7 (−56, 81·7)	169·0 (42·1, 302)
Annual average	Flu	129·0 (79·2, 179)	36·4 (21·6, 51·4)	10·6 (7·5, 13·7)	25·6 (21·3, 29·8)	65·5 (54, 76·9)	125·8 (105, 147)	288·3 (244, 331)
	RSV	1895·8 (1735, 2063)	116·7 (69·6, 167)	1·5 (−9·6, 12·6)	12·1 (−2·1, 26·1)	27·3 (−10·1, 64)	15·3 (−58, 84·6)	174·9 (43·6, 312)

The highest rate of influenza-associated respiratory hospitalizations was among ≥75 year-olds. For RSV, the highest estimate by far was in children age <1 where the rate was an order of magnitude higher than the corresponding one for influenza. A significant burden of RSV-associated respiratory hospitalizations was also found among children 1–4 (with the average rate of RSV-associated hospitalizations estimated to be 3·2 times as high as the corresponding rate for influenza-associated hospitalizations) as well as for ages ≥75.

#### Pneumonia and Influenza hospitalizations

Table2 presents the annual, as well as the average annual estimates for influenza- and RSV-associated P&I hospitalization rates. Tables1 and 2 suggest that most influenza-associated respiratory hospitalizations among children were P&I hospitalizations. Tables1 and 2 combined with data on RSV bronchiolitis hospitalizations estimate that each hospitalization coded as RSV bronchiolitis for age <1 corresponds to 0·37 (0·27, 0·50) RSV-associated non-bronchiolitis respiratory hospitalizations in that age group, of which 0·13 (0·08, 0·18) are P&I hospitalizations. Over the whole period, the rate of RSV-associated P&I hospitalizations is 1·61 times as high as the rate of influenza-associated P&I hospitalizations for age <1 and 3·13 times as high for ages 1–4.

**Table 2:** Estimated annual and average annual rates per 100 000 of influenza- and RSV-associated P&I hospitalizations in different age groups, 2003–2011

Season/Age group (years)		<1	1–4	5–17	18–49	50–64	65–74	≥75
2003–2004	Flu	278·3 (253, 306)	72·6 (59·1, 86·5)	7·0 (3·6, 10·4)	11·1 (9·3, 13·2)	27·3 (20·7, 34·4)	73·3 (58·2, 87)	211·8 (180, 247)
	RSV	186·4 (114, 260)	78·5 (42, 116)	−1·4 (−10·8, 8·1)	6·7 (1·2, 12·3)	6·7 (−10·5, 24·9)	4·8 (−35, 41·5)	62·0 (−27, 156)
2004–2005	Flu	140·1 (101, 178)	−4·3 (−24·4, 16·2)	2·4 (−2·6, 7·4)	11·5 (8·6, 14·4)	38·8 (29·4, 48·9)	117·3 (97·6, 139)	353·0 (302, 408)
	RSV	151·3 (92·9, 211)	63·7 (34·1, 93·9)	−1·2 (−8·8, 6·6)	5·5 (1, 10)	5·4 (−8·5, 20·2)	3·9 (−28·4, 33·7)	50·4 (−21·9, 127)
2005–2006	Flu	55·1 (28·9, 80·8)	16·1 (2·7, 30·4)	3·5 (0·2, 6·9)	5·3 (3·4, 7·3)	17·3 (10·5, 23·8)	38·4 (24·6, 52·4)	108·4 (74, 144)
	RSV	175·1 (108, 244)	73·8 (39·5, 109)	−1·3 (−10·2, 7·6)	6·3 (1·1, 11·6)	6·3 (−9·9, 23·4)	4·5 (−32·9, 39)	58·3 (−25·4, 146)
2006–2007	Flu	57·3 (34·3, 79·9)	10·1 (−1·4, 21·7)	3·9 (1, 6·9)	7·2 (5·6, 8·9)	14·4 (8·5, 20·4)	32·8 (20·6, 45·3)	77·8 (46·6, 107)
	RSV	183·4 (113, 255)	77·3 (41·4, 114)	−1·4 (−10·6, 8)	6·6 (1·2, 12·1)	6·6 (−10·3, 24·5)	4·8 (−34·4, 40·8)	61·0 (−26·6, 153)
2007–2008	Flu	86·0 (49·6, 123)	13·9 (−4·5, 32·9)	5·1 (0·5, 10·0)	10·4 (7·7, 13·1)	23·8 (14·6, 33·7)	52·4 (33, 73·1)	145·3 (94·9, 194)
	RSV	140·8 (86·4, 196)	59·3 (31·8, 87·4)	−1·0 (−8·2, 6·1)	5·1 (0·9, 9·3)	5·1 (−7·9, 18·8)	3·7 (−26·4, 31·3)	46·9 (−20·4, 118)
2008–2009	Flu	53·8 (24·6, 83)	5·0 (−9·7, 20)	3·6 (0·1, 7·2)	7·7 (5·6, 9·9)	11·8 (4·5, 19·3)	27·1 (12, 43·1)	57·9 (19, 95·5)
	RSV	157·5 (96·7, 219)	66·3 (35·5, 97·7)	−1·2 (−9·1, 6·8)	5·7 (1, 10·4)	5·6 (−8·9, 21)	4·1 (−29·6, 35)	52·4 (−22·8, 132)
Spring/Summer 2009	Flu	143·8 (122, 165)	51·9 (40·6, 63·9)	29·6 (26·9, 32·4)	16·2 (14·6, 18)	25·6 (20·3, 31·5)	22·0 (10·6, 33·8)	13·8 (−14, 42·9)
	RSV	6·1 (3·5, 8)	2·4 (1·3, 3·5)	0 (−0·3, 0·2)	0·2 (0, 0·4)	0·2 (−0·3, 0·8)	0·1 (−1·1, 1·3)	1·9 (−0·8, 4·8)
2009–2010	Flu	31·0 (−37·2, 100)	22·3 (0·3, 43·9)	9·5 (3·6, 15·2)	12·1 (8·9, 15·4)	20·1 (9·1, 31·6)	25·2 (2, 49·9)	−1·9 (−59·2, 55·6)
	RSV	257·3 (146, 372)	84·0 (45, 124)	−1·5 (−11·6, 8·7)	7·2 (1·3, 13·2)	7·2 (−11·2, 26·6)	5·2 (−37·5, 44·4)	66·4 (−28·9, 167)
2010–2011	Flu	66·4 (13·8, 120)	−4·2 (−23·6, 16·6)	4·1 (−1·1, 9·4)	9·9 (7, 12·9)	32·1 (21·9, 42·5)	50·5 (29·8, 71·5)	160·6 (107, 217)
	RSV	212·5 (121, 307)	69·4 (37·2, 102)	−1·3 (−9·6, 7·2)	6·0 (1, 10·9)	5·9 (−9·3, 22)	4·3 (−30·9, 36·7)	54·8 (−23·9, 138)
Annual average	Flu	114·0 (93·2, 135)	22·9 (12·8, 33·4)	8·6 (6·1, 11·1)	11·4 (10, 12·9)	26·4 (21·6, 31·4)	54·9 (44·7, 65·2)	140·8 (115, 167)
	RSV	183·8 (115, 254)	71·9 (38·5, 106)	−1·3 (−9·9, 7·4)	6·2 (1·1, 11·3)	6·1 (−9·6, 22·8)	4·4 (−32·0, 38·0)	56·8 (−24·7, 143)

#### Chronic lower respiratory disease hospitalizations

Table3 presents the annual, as well as the average annual estimates for influenza- and RSV-associated CLRD hospitalization rates among adults. Table3 shows a significant contribution of influenza to the burden of CLRD hospitalizations among adults. We estimated that 51·2% of respiratory RSV-associated hospitalizations for ages ≥75 were hospitalizations for CLRD (with the corresponding share for influenza being 30·2%).

**Table 3:** Estimated annual and average annual rates per 100 000 of influenza- and RSV-associated CLRD hospitalizations in the adult age groups, 2003–2011

Season/Age group (years)		18–49	50–64	65–74	≥75
2003–2004	Flu	9·4 (5·7, 13)	35·8 (27·5, 44·2)	58·0 (44·9, 70·9)	82·6 (63·8, 102)
	RSV	1·4 (−8·3, 10·8)	7·1 (−15·5, 29·5)	−0·6 (−37·9, 36·8)	98·4 (47·4, 153)
2004–2005	Flu	17·3 (12·2, 22·6)	60·6 (47·7, 72·7)	125·3 (107, 144)	215·2 (186, 244)
	RSV	1·2 (−6·8, 8·8)	5·7 (−12·6, 24)	−0·5 (−30·8, 29·9)	79·9 (38·5, 124)
2005–2006	Flu	11·0 (7·5, 14·5)	15·6 (7·1, 23·7)	44·0 (31·4, 57)	80·4 (61·6, 99·6)
	RSV	1·3 (−7·8, 10·1)	6·6 (−14·6, 27·7)	−0·5 (−35·6, 34·6)	92·4 (44·6, 144)
2006–2007	Flu	10·3 (7·3, 13·3)	17·8 (10·5, 25)	37·6 (27·1, 48·6)	60·1 (43·3, 77·4)
	RSV	1·4 (−8·2, 10·6)	7·0 (−15·3, 29·1)	−0·5 (−37·3, 36·3)	96·8 (46·7, 150)
2007–2008	Flu	14·6 (9·6, 19·5)	25·3 (13·5, 37)	61·7 (44·7, 79·5)	116·1 (89·5, 144)
	RSV	1·1 (−6·3, 8·2)	5·3 (−11·7, 22·3)	−0·4 (−28·7, 27·8)	74·3 (35·8, 116)
2008–2009	Flu	8·7 (5, 12·6)	17·4 (8·3, 26·7)	31·6 (18·3, 45·6)	48·3 (26·8, 69·7)
	RSV	1·2 (−7, 9·1)	6·0 (−13·1, 25)	−0·5 (−32·1, 31·1)	83·1 (40·1, 129)
Spring/summer 2009	Flu	8·5 (5·7, 11·6)	21·1 (14·2, 27·7)	23·9 (13, 35)	10·0 (−5·9, 25·7)
	RSV	0 (−0·3, 0·3)	0·2 (−0·5, 0·9)	0 (−1·2, 1·1)	3·0 (1·5, 4·7)
2009–2010	Flu	0·9 (−4·9, 7·1)	2·6 (−11·2, 16·9)	8·4 (−12·7, 29·6)	−30·4 (−63·6, 2·6)
	RSV	1·5 (−8·9, 11·6)	7·6 (−16·6, 31·6)	−0·6 (−40·6, 39·4)	105·3 (50·8, 164)
2010–2011	Flu	5·0 (−0·3, 10·6)	33·6 (20·7, 46·3)	46·1 (26·7, 65·5)	115·3 (85·7, 145)
	RSV	1·3 (−7·4, 9·5)	6·3 (−13·7, 26·1)	−0·5 (−33·5, 32·6)	87·0 (41·9, 135)
Annual average	Flu	10·7 (8·1, 13·4)	28·7 (22·5, 34·9)	54·6 (45·4, 64)	87·2 (73, 101)
	RSV	1·3 (−7·6, 9·9)	6·5 (−14·2, 27·0)	−0·5 (−34·7, 33·7)	90·0 (43·4, 139·9)

### Non-respiratory causes

Table4 records average annual estimates of influenza- and RSV-associated hospitalization rates with several non-respiratory principal diagnoses.

**Table 4:** Estimated average annual estimates for rates per 100 000 of influenza- and RSV-associated hospitalizations with select principal diagnosis categories, 2003–2011

Diagnosis/Age group (years)		<1	1–4	5–17	18–49	50–64	65–74	≥75
Circulatory	Flu	8·0 (−7·2, 22·9)	−1·1 (−6·1, 4·0)	0 (−1·3, 1·2)	−2·9 (−6·1, 0·2)	−19·9 (−36·1, −3·5)	−40·2 (−81·2, 0·04)	15·9 (−38·3, 71·3)
	RSV	−9·4 (−60·3, 43·3)	−8·1 (−26·7, 10·0)	−0·3 (−4·7, 4·1)	3·4 (−7·0, 14·3)	37·0 (−23·6, 91·8)	−28·2 (−170·9, 111·7)	199·1 (12·5, 375·4)
Diabetes	Flu	0	0	0·4 (−1, 1·9)	0·1 (−1·1, 1·4)	−3·3 (−6·9, 0·3)	−1·4 (−9·2, 6·3)	1·5 (−7·2, 10·5)
	RSV	0	0	−2·8 (−7·9, 1·9)	0·7 (−3·8, 5·1)	12·6 (−0·3, 25·9)	−4·4 (−32·3, 23·6)	−9·3 (−41·5, 23·3)
Renal disease	Flu	0	−0·5 (−0·8, 0)	−0·3 (−1, 0·4)	0·1 (−0·4, 0·7)	−0·5 (−2·7, 1·9)	2·3 (−3, 7·4)	10·8 (−0·2, 22·3)
	RSV	0	1·1 (−0·2, 2·7)	0·4 (−1·9, 3)	−1·3 (−3·3, 0·7)	−0·4 (−8·8, 7·6)	2·1 (−16·9, 22·1)	3·4 (−34, 41·8)
Alzheimer’s disease	Flu	0	0	0	0	0	−2·4 (−5·2, 0·5)	0·2 (−4·1, 4·4)
	RSV	0	0	0	0	0	−8·6 (−18·8, 1·6)	0·9 (−14·8, 16·8)
Septicemia	Flu	0·4 (−10·9, 11·9)	0 (−0·5, 0·7)	0·8 (0·1, 1·4)	1·0 (0·3, 1·7)	4·0 (1·7, 6·3)	8·8 (2·2, 15·6)	38·7 (25·7, 52·9)
	RSV	30·5 (−10·2, 71·3)	0·1 (−1·9, 2·4)	−1·1 (−3·2, 1·1)	1·6 (−0·8, 4·1)	1·3 (−6·9, 9·4)	3·2 (−21·1, 27·4)	−5·7 (−57·9, 42·6)

We note that the estimates of both influenza’s and RSV’s contribution in our model do not attain statistical significance for most categories of hospitalization we have considered. At the same time, Table4 suggests a significant contribution of influenza to septicemia hospitalizations for ages >5, and a high rate of RSV-associated circulatory hospitalizations for ages ≥75.

## Discussion

Considerable uncertainty exists regarding the burden of hospitalization associated with influenza and RSV. We have applied a linear regression scheme to hospitalization data in NYC with incidence proxies for the major influenza subtypes and RSV, combined with a flexible model for the baseline of hospitalizations not associated with influenza or RSV, to estimate the burden for a variety of principal diagnoses of hospitalization in different age groups between 2003 and 2011. We found a significant contribution of influenza to the rates of respiratory and P&I hospitalizations in all age groups, as well as CLRD hospitalizations in adults and septicemia hospitalizations for ages ≥5. RSV made a very high contribution to the rates of respiratory hospitalizations for age <1 and a substantial contribution to the rates of respiratory hospitalizations for ages 1–4, P&I hospitalizations for ages 18–49 as well as all respiratory, CLRD, and circulatory hospitalizations for ages ≥75.

Our estimates are generally compatible with those in the literature.^11^,^22^ In particular, we have found that influenza-associated hospitalization rates are highest in the oldest age groups, while the RSV-associated hospitalization rate is highest for age <1 and an order of magnitude higher than the corresponding rate for influenza. We have also found a sizeable contribution of RSV to both the respiratory and circulatory hospitalization burden for ages ≥75 with much smaller estimates lacking significance for ages 65–74. Our estimates for the rates of RSV-associated hospitalizations among the elderly are higher than those in the literature,^11^,^22^ with further work needed to assess the fiscal burden associated with RSV infections in the elderly. We also note that while most estimates in the literature of the burden of severe outcomes associated with RSV among the elderly are given for ages ≥65, some evidence exists that this burden is skewed heavily toward the oldest adults (e.g., a median age of 80 among RSV-positive hospitalized adults ≥18 in ref.^4^). Moreover, we have found that hospitalizations with principal diagnoses of CLRD and circulatory causes are relatively more prominent among RSV-associated hospitalizations for ages ≥75 compared with influenza-associated hospitalizations, suggesting that adults aged ≥75 with heart or chronic lower respiratory disease are potential priority groups for an RSV vaccine.

The rates of respiratory hospitalizations corresponding to the antigenically novel Fujian A/H3N2 strain during the 2003–2004 season in children were higher than the rates during the subsequent 2004–2005 season, while the opposite was true for adults. An analogous result for ED visits was presented in ref.^23^ We also note that the corresponding age discrepancy was detected for the pandemic year versus the 2010–2011 A/H1N1 season in other settings,^24^,^25^ with a high burden in adults during the latter season. These observations accord with the notion that novel influenza strains cause disproportionate infection among children with the resulting immunity in that population contributing to the change in the age distribution of cases during the next year’s influenza season. We also note that the 2010–2011 season in NYC had minimal A/H1N1 activity and no analogous age discrepancy was discerned for the spring and the fall waves of the 2009 pandemic, suggesting that perhaps the spring wave was incomplete in terms of the depletion of susceptibles among children, possibly stalled by the school break and the summer weather, with the fall wave serving largely as a continuation of the spring wave.

A trigonometric model for the baseline rates of hospitalizations not associated with influenza and RSV is commonly employed.^11^ In the Supporting information, we have examined the performance of the model analogous to the Main Inference Method (eq. 1) with the baseline assumed to be a linear combination of the sine and cosine functions. We have found the Main Inference Method consistently exhibits a better fit (lower AIC score). Moreover, the estimates of the two models can be different, particularly for RSV-associated hospitalizations, suggesting that the rigid assumption that the baseline model is trigonometric may not be applicable in certain contexts, biasing the estimation of the severe outcome burden associated with respiratory pathogens.

Our study has several limitations. This analysis was based on the 2003–2011 data for NYC and might to some extent reflect local respiratory virus testing and hospitalization coding practices, as well as the contribution from influenza strains circulating during that time period. Another possible limitation is the potential temporal inconsistency between our influenza and RSV incidence proxies and their associated hospitalizations rates. For influenza, we have attempted to rectify this as explained in the Supporting information. For RSV, the proposed linear relation between the rates of hospitalizations coded for RSV bronchiolitis and RSV incidence hinges on the high specificity of the RSV bronchiolitis diagnosis as suggested in the literature.^26^–^28^ We further examined our RSV incidence proxy in the Supporting information in relation to viral testing data and compared its performance in fitting the hospitalization data relative to other possible incidence proxies. Yet another potential source of uncertainty is the suitability of the proposed inference model to the various hospitalization datasets. Poor suitability might arise from the fact that rates of hospitalization not associated with influenza and RSV might not correspond to a combination of a periodic baseline and temporal trend. For example, we removed asthma and croup hospitalizations from pediatric respiratory counts due to their aperiodicity to obtain a better model fit and did not present the model’s estimates for all-cause hospitalizations due to uncertainty about the aperiodic factors. This issue might be more severe for influenza than for RSV, with one RSV incidence proxy compared with eight for influenza (due to the presence of the major subtypes, effects of the 2009 A/H1N1 pandemic, apparent changes in the age distribution of severe outcomes for A/H3N2, etc., as indicated in the Supporting information). Those finely granulated influenza incidence proxies might become spuriously associated with rates of hospitalizations not caused by influenza or RSV that do not accord with the baseline plus trend structure during certain time periods. This might be the reason behind the model’s inability to ascertain the rates of influenza-associated hospitalizations with a number of non-respiratory principal diagnoses (particularly circulatory causes, with a negative estimate for ages 50–64 in our model) for which a positive contribution of influenza to mortality was established.^13^,^29^ At the same time, it is also possible that influenza rather than RSV infections in individuals with underlying heart conditions cause upper respiratory symptoms that result in those hospitalizations being coded as respiratory rather than circulatory, while deaths stemming from those hospitalizations are caused by complications associated with the heart disease and are coded as circulatory. Further work will be required with additional datasets to assess the association of influenza with all-cause hospitalizations and hospitalizations with a principal diagnosis of circulatory disease.

In summary, despite some limitations, our inference methodology provides a consistent relation between the incidence of influenza and RSV and the associated hospitalization outcomes, a relation that is linear (proportional) and avoiding parametric assumptions about the weekly hospitalization rates not associated with the two pathogens. The results in our study contribute to the understanding of the hospitalization burden associated with influenza and respiratory syncytial virus, stressing the need for the mitigation of the impact of the annual epidemics corresponding to those two respiratory viruses, including the possibility of developing a vaccine for RSV.^3^,^30^

## Supporting Information

**Appendix S1**. ICD-9-CM codes for the hospitalization categories used in the analyses. **Appendix S2**. Incidence proxies for influenza **Appendix S3**. Incidence proxy for RSV. **Appendix S4**. Model fits for respiratory hospitalizations **Appendix S5**. R-squared for the main inference model **Appendix S6**. Trigonometric model for the baseline. **Appendix S7**. More on the selection of the influenza incidence proxies. **Appendix S8**. Alternative inference methods.
