# Supplementary material for: Estimating the hospitalization burden associated with influenza and respiratory syncytial virus in New York City, 2003–2011
Source: Influenza Other Respir Viruses. 2015 Aug 4;9(5):225–33. doi: 10.1111/irv.12325 (PMC4548992; doi:10.1111/irv.12325)
Supplement: Supplementary file 1 [file irv0009-0225-sd1.doc]

Appendix for “**Estimating the hospitalization burden associated with influenza and respiratory syncytial virus in New York City, 2003-2011**”

**Appendix S1. ICD-9-CM codes for the hospitalization categories used in the analyses**

| **Category** | **Principal ICD-9-CM diagnosis codes** | **Remarks** |
| --- | --- | --- |
| All respiratory | 460-519 | For ages 0-17, asthma (ICD-9-CM 493) and croup (ICD-9-CM 464.4) hospitalizations were excluded |
| Pneumonia and Influenza (P&I) | 480-487 (2003-2008)  480-488 (2009-2011) |  |
| Chronic lower respiratory disease | 490-494; 496 | For ages 0-17, asthma (ICD-9-CM 493) hospitalizations were excluded |
| Circulatory | 390-459 |  |
| Diabetes | 250 |  |
| Renal disease | 580-589 |  |
| Alzheimer’s disease | 331.0 |  |
| Septicemia | 038 |  |
| RSV bronchiolitis | 466.11 | Rates for age <1 used as incidence proxy for RSV |

**Appendix S2. Incidence proxies for influenza**

Following , for each major influenza (sub)type (A/H3N2, A/H1N1 and B), we defined the weekly incidence proxy for that subtype to be the percent of emergency department (ED) visits in NYC classified as influenza-like illness (ILI) multiplied by the percent of respiratory specimens which were positive for a given subtype. To estimate the latter percentages and adjust for potential temporal inconsistency between the above proxies and the rates of hospitalizations associated with the corresponding influenza subtypes (a temporally varying ratio of those two quantities for certain combinations of an influenza subtype/age group), the following conventions were adopted:

(i) Given the lack of sub-typing for influenza A specimens in the NYC Department of Health and Mental Hygiene (DOHMH) active laboratory surveillance data, the corresponding weekly data for the NY state (provided by the Lyn Finelli / US CDC) were utilized, with the percent of A/H3N2 and A/H1N1 specimens among the NYC DOHMH influenza A samples assumed to be proportional to the numbers of A/H3N2 and A/H1N1 specimens in the NY state data as in .

(ii) For influenza A/H1N1, information on whether the specimen in the NY state data represented a seasonal or pandemic strain was available, and correspondingly the incidence proxy was split into two – seasonal and pandemic (starting week 16, 2009). Henceforth by “splitting” the incidence proxy into two periods we mean that the proxy is replaced by two proxies: the first proxy equals the original proxy during the first time period, and zero during the second time period, with the opposite being true for the second incidence proxy. Moreover, visual examination of the ILI levels during the spring wave of the pandemic (Figure S1) suggests disproportionately high levels of ILI during the late spring/early summer of 2009 compared with influenza circulation levels in the population relative to other seasons – such a discrepancy might stem from an increased likelihood of seeking medical services for ILI during the nascent pandemic compared with ILI cases during regular flu seasons . Therefore the pandemic A/H1N1 incidence proxy was split into two: Spring/Summer 2009 (weeks 16-34, 2009) and the subsequent incidence.

(iii) Temporal changes in the relation between the incidence proxy for influenza A/H3N2 and the rates of A/H3N2-associated hospitalizations in various age groups are likely. For example, the 2003-2004 season carried an exceptionally high burden of A/H3N2-associated severe outcomes among young children (as suggested by our results, as well as ), while the 2004-2005 season had an exceptionally high burden of A/H3N2-associated severe outcomes among the elderly. The latter likely stems from both the difference in the age-distributions for the A/H3N2 cases during the 2003-2004 season compared with the 2004-2005 season (with our incidence proxy incorporating no age-specific information) as well as temporal changes in A/H3N2-associated case-hospitalization rates in certain age groups. In addition, changes in the virological surveillance following the 2009 A/H1N1 pandemic are expected to entail a change in the relation between our incidence proxies and the population incidence for each influenza subtype for the post-pandemic period compared with the pre-pandemic period. To accommodate those potential changes, the incidence proxy for influenza A/H3N2 was split into four time periods – 2003-2004 season, 2004-2005 season, 2006-2007 through 2008-2009 seasons (pre-pandemic period) and the post-pandemic period. We also note that little influenza B circulation took place after the 2009 pandemic in NYC through 2011 (Figure A2), and the corresponding splitting for the influenza B incidence proxy wasn’t warranted. Figure S2 depicts the incidence proxies for influenza A/H3N2, A/H1N1 and B, with their splitting described above used in the inference.

**Figure S1:** Weekly percentages of emergency department visits classified as ILI in NYC between 2003-2011

**Figure S2:** Incidence proxies for influenza A/H3N2 (red), A/H1N1 (blue) and B (green), NYC, 2003-2011

**Appendix S3. Incidence proxy for RSV**

We used weekly rates of hospitalizations coded for RSV bronchiolitis for age <1 as an RSV incidence proxy. This choice is premised on previous estimates suggesting that the majority (about 70% ) of bronchiolitis cases among young children correspond to RSV infections. Moreover, splitting of the bronchiolitis diagnosis into RSV-associated (ICD-9-CM 466.11) and other (ICD-9-CM 466.19), which for most US children admitted to a hospital with a bronchiolitis diagnosis is supported by diagnostic tests to identify RSV infection , should further improve the specificity of this data stream. Figure S3 exhibits the weekly rates of RSV bronchiolitis, as well as all respiratory hospitalizations (with asthma and croup excluded) for age <1.

**Figure S3:** Weekly rates (per 100,000) of RSV bronchiolitis (red) and all respiratory hospitalizations (black) for age <1, NYC 2003-2011

We see that unlike the ILI rates (Figure S1), rates of RSV bronchiolitis hospitalization taper off during the summer, suggesting a higher specificity of the latter diagnosis . To further examine the above incidence proxy, we compared it with the data on RSV testing of respiratory specimens. These data between 2003-2011 were split into three time periods according to its source:

(1) 2003-2006 US HHS region 2 data (provided by Amber Haynes / US CDC) on the percent of specimens testing positive for RSV

(2) 2006-2009 NY state data (provided by Amber Haynes / US CDC) on the percent of specimens testing positive for RSV

(3) 2009-2011 NYC active laboratory surveillance data (provided by Beth Nivin / NYC DOHMH) on the percent of specimens testing positive for RSV

Figure S4 plots the weekly rates of RSV bronchiolitis for age <1 in NYC against the percent of respiratory specimens testing positive for RSV as described above. It appears visually that testing was reasonably consistent between 2006-2009 (period (2)). The correlation between the percent positive for RSV and our incidence proxy during that time period is 0.925(0.9,0.94).

**Figure S4:** Weekly rates (scaled) of RSV bronchiolitis for age <1 in NYC (black) and the percent of respiratory specimens testing positive for RSV as described in (1-3) (red).

We examined the possibility of using other incidence proxies for RSV by comparing the model fit for our analysis for respiratory hospitalizations for age <1 as well as hospitalizations with underlying P&I (the latter categories were chosen due to the high contribution of RSV – see Figure S3 as well as Tables 1 and 2 in the main text), with the results presumably sensitive to the choice of the RSV incidence proxy. The alternative RSV incidence proxies we considered were

1. Percent of respiratory specimens testing positive for RSV (split into the 2003-2009 and 2009-2011 periods as suggested by Figure A4).
2. Rate of RSV bronchiolitis for age <1 olds multiplied by the percent of respiratory specimens testing positive for RSV (split into the 2003-2009 and 2009-2011 periods).

Table S1 shows the Akaike Information Criterion (AIC) scores for the corresponding model fits. We see that while multiplying the percent of specimens positive for RSV by rates of RSV bronchiolitis for age <1 results in better model fits compared with the proxies using percent positive for RSV alone, the RSV incidence proxy used in the main analysis provides the lowest AIC score for both the respiratory and the P&I hospitalization data.

| Type /RSV proxy | % RSV positive | % RSV positive times  RSV bronchiolitis rate | RSV bronchiolitis rate |
| --- | --- | --- | --- |
| All respiratory | 3429.6 | 3261.4 | 3017.1 |
| P&I | 2403.6 | 2395.7 | 2381.7 |

**Table S1:** AIC scores for the model fits for all respiratory and P&I hospitalizations for age <1 with different RSV incidence proxies – those given by (A) and (B) (columns 1 and 2) and the incidence proxy used in the main analysis (column 3).

**Appendix S4. Model fits for respiratory hospitalizations**

Figures S5-S9 plot the model fits as well as the baseline rates for hospitalizations not associated with influenza and RSV for the categories of all respiratory, pneumonia & influenza (P&I) and chronic lower respiratory disease hospitalizations in the different age groups. Those figures exhibit temporally consistent model fits for all the age groups and principal diagnoses considered. In particular, that refers to the spikes of varying magnitude (depending on the age groups, season, and principal diagnosis) during the 2003-2004 and 2004-2005 seasons driven by influenza A/H3N2 (Figure S2); the spike among children ages <1 and 1-4 during the 2006-2007 season, presumably driven by the exceptionally strong RSV epidemic (Figure S3); the spikes among adults during the 2007-2008 season, presumably driven by a strong influenza season with a mixture of various subtypes (Figure S2); the spring wave of the 2009 A/H1N1 pandemic in the non-elderly age groups; the Fall wave of the 2009 A/H1N1 pandemic, particularly for ages 1-4; and the strong 2010-2011 A/H3N2 season (Figure S2), particularly for adults ages over 50.

***All respiratory hospitalizations***

**Figure S5:** Weekly rates per 100,000 of respiratory hospitalizations among children (black), model fits (red), and the baseline + trend (blue).

**Figure S6:** Weekly rates per 100,000 of respiratory hospitalizations among adults (black), model fits (red), and the baseline + trend (blue).

***Pneumonia and Influenza (P&I) hospitalizations***

**Figure S7:** Weekly rate per 100,000 of P&I hospitalizations among children (black), model fits (red), and the baseline + trend (blue).

**Figure S8:** Weekly rate per 100,000 of P&I hospitalizations among adults (black), model fits (red), and the baseline + trend (blue).

***Chronic lower respiratory disease (CLRD) hospitalizations***

**Figure S9:** Weekly rate per 100,000 of CLRD hospitalizations among adults (black), model fits (red), and the baseline + trend (blue).

**Appendix S5. R-squared for the Main Inference Model**

Table S2 exhibits the R-squared estimates for the Main Inference Method (eq. 1) for various principal hospitalization diagnoses in the different age groups. We note that those R-squared estimates partly reflect the noise coming from the hospitalization count data; thus cross-comparison of the R-squared estimates for the various hospitalization diagnoses/age groups may be dubious.

|  | <1 | 1-4 | 5-17 | 18-49 | 50-64 | 65-74 | ≥75 |
| --- | --- | --- | --- | --- | --- | --- | --- |
| Respiratory | 0.98 | 0.91 | 0.73 | 0.85 | 0.81 | 0.83 | 0.89 |
| P&I | 0.86 | 0.87 | 0.78 | 0.82 | 0.74 | 0.79 | 0.89 |
| CLRD | 0 | 0 | 0 | 0.77 | 0.76 | 0.76 | 0.8 |
| Circulatory | 0.04 | 0.12 | 0.04 | 0.47 | 0.56 | 0.61 | 0.61 |
| Diabetes | 0 | 0 | 0.06 | 0.22 | 0.3 | 0.37 | 0.38 |
| Renal Disease | 0 | 0 | 0.16 | 0.21 | 0.41 | 0.34 | 0.3 |
| Alzheimer | 0 | 0 | 0 | 0 | 0 | 0.37 | 0.65 |
| Septicemia | 0.08 | 0.05 | 0.21 | 0.8 | 0.85 | 0.75 | 0.86 |

**Table S2:** R-squared for the OLS fits in the Main Inference Method for various principal hospitalization diagnoses and age groups.

**Appendix S6. Trigonometric model for the baseline**

For our main inference method, we modeled the baseline of the weekly rates (per 100,000) of hospitalization outcomes not associated with influenza or RSV by periodic cubic splines to accommodate for its a-priori unknown shape. Traditionally, this baseline is assumed to follow a trigonometric model (a linear combination of the sine and the cosine functions with a period of one year). Here, we consider the performance of the corresponding model, namely the one is analogous to the Main Model (eq. 1) with the baseline assumed to be a linear combination of the sine and cosine functions. We compare the performance of this model to the performance of the main model (both in terms of the AIC score for the model fits as well as the model estimates) for respiratory hospitalizations in different age groups. Table S3 summarizes this comparison:

|  | | <1 | 1-4 | 5-17 | 18-49 | 50-64 | 65-74 | ≥75 |
| --- | --- | --- | --- | --- | --- | --- | --- | --- |
| Main Model | AIC | 3032.3 | 2013.6 | 849 | 943.1 | 1766.1 | 2330.7 | 2787.7 |
|  | Flu | 129.0  (79.2,179) | 36.4  (21.6,51.4) | 10.6  (7.5,13.7) | 25.6  (21.3,29.8) | 65.5  (54,76.9) | 125.8  (105,147) | 288.3  (244,331) |
|  | RSV | 1895.8  (1735,2063) | 116.7  (69.6,167) | 1.5  (-9.6,12.6) | 12.1  (-2.1,26.1) | 27.3  (-10.1,64) | 15.3  (-58,84.6) | 174.9  (43.6,312) |
| Trig. Baseline  Model | AIC | 3087.9 | 2225.6 | 937.3 | 1039.3 | 1813.5 | 2397.5 | 2862.1 |
|  | Flu | 95.5  (39.6,156.4) | 22.4  (-2.3,44.9) | 8.3  (4.2,12.3) | 21.5  (17,26.9) | 57.2  (45,70.4) | 109.9  (82.6,133.9) | 258.7  (204.6,310.4) |
|  | RSV | 1988.1  (1896,2085) | 162.3  (119.9,210.3) | 2.2  (-5.7,10) | -4.7  (-14.2,4.5) | 4.3  (-19.4,26.9) | 21.8  (-27.1,67.8) | 67.3  (-24,160.3) |

**Table S3:** AIC scores and estimated average annual rates of influenza and RSV-associated respiratory hospitalizations per 100,000 in different age groups for the Main Model vs. a model with a trigonometric baseline

We see that the main model consistently exhibits a better fit (lower AIC score). Moreover the estimates of the two models can be different, particularly for RSV-associated hospitalizations. The latter may be partly explained by the high year-to-year periodicity of RSV circulation compared to influenza (Figure S2 vs. S4), which could confound the estimation of RSV-attributable outcomes if the true baseline varies in a fashion correlated with RSV.

**Appendix S7. More on the selection of the influenza incidence proxies**

In applying our main inference method, we split the incidence proxies for the major influenza (sub)types into several time periods (Appendix S2) to reflect various changes in the relation between influenza incidence and its ascertainment in the NYC surveillance systems. Thus the A/H3N2 proxy was split into h31 (equaling the A/H3N2 incidence proxy during the 2003-2004 season and zero outside this season), h32 (2004-2005), h33 (2005-2008) and h34 (2010-2011); additionally, the A/H1N1 incidence proxy was split into h11 (2003-February 2009), h12 (Spring-Summer 2009) and h13 (Fall 2009 -2011). The logical justification behind that splitting is presented in Appendix S2. Here we provide additional statistical support for this splitting, considering the performance of the main inference method for respiratory hospitalizations in each age group when pairs of temporally consecutive incidence proxies that we’ve considered for either influenza A/H3N2 or A/H1N1 (e.g. h32 and h33 or h11 and h12) are joined together. Table S4 depicts the AIC score for the various models and age groups

|  | <1 | 1-4 | 5-17 | 18-49 | 50-64 | 65-74 | ≥75 |
| --- | --- | --- | --- | --- | --- | --- | --- |
| M1 (h31+h32) | 3037.9 | 2027.8 | 848.5 | 991.1 | 1846.6 | 2469.8 | 3039.9 |
| M2 (h32+h33) | 3034.3 | 2012.2 | 849.4 | 942.1 | 1777.4 | 2344.8 | 2831.3 |
| M3 (h33+h34) | 3030.7 | 2011.7 | 847.2 | 950.9 | 1764.4 | 2340.5 | 2795.1 |
| M4 (h11+h12) | 3031.2 | 2012.5 | 850.6 | 970.9 | 1770.2 | 2334.7 | 2788.6 |
| M5 (h12+h13) | 3030.4 | 2054.4 | 848.7 | 941.9 | 1764.4 | 2328.9 | 2791.3 |
| Main Model | 3032.3 | 2013.6 | 849 | 943.1 | 1766.1 | 2330.7 | 2787.7 |

**Table S4**: AIC scores for the model fits for respiratory hospitalizations in different age groups

We see that while the main model does not minimize the AIC score for all age groups, compared with each alternative model, the main model offers a significant improvement in the AIC score for some age groups while the opposite is not true for any alternative model vs. the main model. For example, compared with model M1, there is a major improvement for ages 75+, 65-75,50-64, 18-49 and 1-4; compared with M2, there is a major improvement for ages 75+,65-74, 50-64; compared with M3, there is a major improvement for ages 65-74, 75+ and 18-49; compared with M4, there is a major improvement for ages 18-49, and to a lesser extend, 50-64 and 65-74; compared with M5, there is a major improvement for ages 1-4, and, to a lesser extent, 18-49.

**Appendix S8. Alternative inference methods**

*Method A1*

In this section, we consider the following model: For each principal diagnosis category of hospitalization and age group, weekly hospital admission rates (per 100,000 individuals in that age group) were regressed linearly against incidence proxies and for the major influenza subtypes and RSV (see Appendices S2 and S3), a temporal trend (modeled by a low degree polynomial in time) and a seasonal baseline (modeled by periodic cubic splines with a period of one year):

(S1)

Here $S()$ is a forward shift between 0 and 1 weeks reflecting the lag between influenza infection and hospitalization as before, and the noise is assumed to follow an autoregressive AR(1) process. Other than the use of autocorrelated noise, this method is similar to the Main Inference Method except that we use a likelihood-based approach in the framework of eq. S1 to produce the model’s estimates and confidence bounds rather than adopt the OLS estimates. We bootstrap the confidence bounds as in the inference method in the main body of the text. Table S5 exhibits the model’s average annual estimates of the rates of influenza and RSV-associated hospitalization (per 100,000 individuals) with various principal diagnoses in different age groups. We see that those estimates are in a good agreement with the Main Inference Method estimates (Tables 1-4).

|  | | <1 | 1-4 | 5-17 | 18-49 | 50-64 | 65-74 | ≥75 |
| --- | --- | --- | --- | --- | --- | --- | --- | --- |
| Respiratory | Flu | 127.8  (76.1,179.5) | 36.8  (21.6,51.9) | 10.6  (7.4,13.8) | 25  (20.8,29.3) | 62.1  (50.5,73.7) | 123.9  (102.2,145.6) | 283.9  (240.8,327) |
|  | RSV | 1850.3  (1689,2011) | 100  (52.6,147.5) | 2.3  (-8.6,13.3) | 18.3  (5.3,31.2) | 36.9  (1.5,72.2) | 39.8  (-29.2,108.7) | 198  (75.6,320.4) |
| P&I | Flu | 114.4  (92.3,136.6) | 23.8  (13.3,34.3) | 8.9  (6.4,11.4) | 11.4  (9.9,12.9) | 25.4  (20.4,30.5) | 55.3  (44.7,66) | 139.1  (112.9,165) |
|  | RSV | 178.6  (107.1,250) | 57.6  (25,90.2) | -0.8  (-9.2,7.6) | 6.5  (1.4,11.5) | 8.9  (-6.9,24.8) | 11.8  (-22.8,46.4) | 71.8  (-2.3,145.9) |
| CLRD | Flu | 0(0,0) | 0(0,0) | 0(0,0) | 10.6  (7.9,13.3) | 27.7  (21.4,34) | 54.4  (44.6,64.2) | 87.2  (72.3,102) |
|  | RSV | 0(0,0) | 0(0,0) | 0(0,0) | 6.7  (-1.7,15) | 11.4  (-9.1,31.9) | 1  (-33.8,35.8) | 103.2  (54.3,152.1) |
| Circulatory | Flu | 8.0  (-7.0,23.0) | -1.1  (-6.3,4.0) | 0  (-1.3,1.2) | -2.9  (-6.1,0.2) | -20.5  (-37.3,-3.8) | -42  (-83.2,-0.9) | 12.6  (-42.9,68.2) |
|  | RSV | -9.4  (-62.3,43.5) | -8.3  (-27.1,10.4) | -0.6  (-5.2,3.9) | 4  (-6.8,14.8) | 39.1  (-19.7,97.9) | -17.2  (-161,126.5) | 209.5  (20,399.1) |
| Diabetes | Flu | 0(0,0) | 0(0,0) | 0  (-1.5,1.5) | 0.4(-1,1.8) | -4.1  (-11.5,3.3) | -17.8  (-34.6,-1.1) | -17  (-34.1,0.1) |
|  | RSV | 0(0,0) | 0(0,0) | -0.5  (-6,5) | -3.7  (-8.8,1.5) | -7  (-34.6,20.6) | 31  (-29.8,91.7) | -17.4  (-78.1,43.2) |
| Renal  Disease | Flu | 0(0,0) | 0(0,0) | 0.4  (-1.1,1.8) | 0.1  (-1.1,1.4) | -3.3  (-7.1,0.4) | -1.2  (-9.3,6.9) | 1.5  (-7.8,10.8) |
|  | RSV | 0(0,0) | 0(0,0) | -2.5  (-7.5,2.5) | 0.6  (-4,5.2) | 12.4  (-1,25.8) | -3.6  (-32.4,25.1) | -9.2  (-42.7,24.3) |
| Alzheimer | Flu | 0(0,0) | 0(0,0) | -0.3  (-1,0.4) | 0.1  (-0.4,0.7) | 0  (0,0) | 2.3  (-3.2,7.7) | 9.9  (-1.5,21.3) |
|  | RSV | 0(0,0) | 0(0,0) | 0.5  (-2,2.9) | -1.3  (-3.4,0.8) | 0  (0,0) | 2.3  (-18,22.6) | 7.3  (-31.7,46.4) |
| Septicemia | Flu | 0.4  (-11.6,12.4) | 0  (-0.6,0.6) | 0.8  (0.1,1.4) | 1  (0.3,1.7) | 4  (1.6,6.4) | 8.9  (2,15.7) | 38.7  (24.6,52.8) |
|  | RSV | 30.7  (-11.9,73.4) | 0.1  (-1.9,2.1) | -1.4  (-3.6,0.9) | 1.6(-0.9,4) | 1.3  (-7.2,9.8) | 3.4  (-21.5,28.4) | -5.9  (-57.6,45.8) |

**Table S5:** Average annual estimates of the rates of influenza and RSV-associated hospitalization (per 100,000 individuals) with various principal diagnoses in different age groups, model A1

*Method A2*

In this section, we consider the following model: For each principal diagnosis category of hospitalization and age group, weekly hospital admission counts $C(t)$ we modeled as

$C(t)=Poisson(\mu(t))$ (S2)

where

$$\mu(t)=\sum_{i} \beta_{i}^{flu}\cdot S(Flu_{i}(t))+\beta^{RSV}\cdot RSV(t)+Baseline+Trend$$

Thus the noise in this model comes from the noise in the Poisson-distributed counts, and there is no correlation between the noise on different weeks. Table S6 exhibits the model’s average annual estimates of the rates of influenza and RSV-associated hospitalization (per 100,000 individuals) with various principal diagnoses in different age groups. We see that those estimates are in a good agreement with the Main Inference Method estimates (Tables 1-4).

|  | | <1 | 1-4 | 5-17 | 18-49 | 50-64 | 65-74 | ≥75 |
| --- | --- | --- | --- | --- | --- | --- | --- | --- |
| Respiratory | Flu | 130.7  (80.9,180.5) | 37.6  (26.5,48.7) | 10.6  (8.1,13.0) | 25.7  (23.5,27.9) | 66  (59.7,72.3) | 126  (112,140) | 286.1  (264.6,307.6) |
|  | RSV | 1857.1  (1680.1,2034) | 117  (76.9,157.2) | 1.5  (-7.3,10.2) | 12.3  (4.9,19.8) | 25.9  (4.5,47.2) | 10.5  (-37.3,58.3) | 166.6  (94.9,238.2) |
| P&I | Flu | 115.6  (92.8,138.3) | 23.6  (15.6,31.6) | 8.4  (6.6,10.3) | 11.5  (10.3,12.6) | 26.  6(23.2,30) | 55.5  (47.7,63.2) | 140.6  (127.3,153.9) |
|  | RSV | 180.4  (110.6,250.2) | 69.5  (40.9,98) | -1.2  (-7.7,5.2) | 6.1  (2.3,9.9) | 5.4  (-5.8,16.6) | 5.3  (-20.9,31.5) | 51.1  (6.2,96) |
| CLRD | Flu | 0(0,0) | 0(0,0) | -17.2  (-20.1,-14.2) | 10.9  (9.4,12.3) | 29  (24.7,33.3) | 54.4  (45,63.8) | 87.1  (75.2,99.1) |
|  | RSV | 0(0,0) | 0(0,0) | -6.3  (-18.1,5.6) | 1.3  (-3.8,6.4) | 5.3  (-9.6,20.2) | -3.1  (-34.9,28.8) | 88.2  (48.7,127.8) |
| Circulatory | Flu | 0(0,0) | 0(0,0) | -0.1  (-1.1,1) | -2.9  (-5.1,-0.7) | -20.3  (-29.1,-11.5) | -42.2  (-63.3,-21.1) | 17.3  (-12.9,47.4) |
|  | RSV | 0(0,0) | 0(0,0) | -1.2  (-4.8,2.4) | 3.4  (-4.4,11.2) | 36.3  (4.2,68.3) | -26.5  (-103.3,50.4) | 194.9  (87.9,301.9) |
| Diabetes | Flu | 0(0,0) | 0(0,0) | -0.1(-1,0.7) | 0.3  (-0.8,1.5) | -4.4  (-9.1,0.4) | -17.1  (-27.9,-6.4) | -17.6  (-29.8,-5.5) |
|  | RSV | 0(0,0) | 0(0,0) | -0.2  (-3.3,2.9) | -3.5  (-7.7,0.7) | -6.5  (-24.1,11.2) | 32.3  (-7.8,72.5) | -21  (-65.5,23.6) |
| Renal  Disease | Flu | 0(0,0) | 0(0,0) | 0.5  (-0.9,1.8) | 0.1  (-1.1,1.3) | -3  (-6.7,0.6) | -1.2  (-8.5,6.1) | 1.6  (-7,10.2) |
|  | RSV | 0(0,0) | 0(0,0) | -3(-7.7,1.6) | 0.6  (-3.7,5) | 12.5  (-0.3,25.2) | -3.5  (-29.8,22.7) | -9.5  (-40,21) |
| Alzheimer | Flu | 0(0,0) | 0(0,0) | 0(0,0) | 0  (0,0) | 0  (0,0) | 2  (-2.9,6.9) | 9.7  (1.5,17.9) |
|  | RSV | 0(0,0) | 0(0,0) | 0(0,0) | 0  (0,0) | 0  (0,0) | 2.1  (-15.4,19.6) | 3.7  (-24.9,32.2) |
| Septicemia | Flu | 0(0,0) | 0(0,0) | 0(0,0) | 0.9  (0.2,1.5) | 4.2  (1.6,6.7) | 8.7  (2,15.4) | 39.1  (26.1,52.2) |
|  | RSV | 0(0,0) | 0(0,0) | 0(0,0) | 1.4  (-0.7,3.5) | 0.2  (-8.2,8.6) | 2.7  (-20.7,26.1) | -10.4  (-55.4,34.6) |

**Table S6:** Average annual estimates of the rates of influenza and RSV-associated hospitalization (per 100,000 individuals) with various principal diagnoses in different age groups, model A2.

**References**

1. Goldstein E, Viboud C, Charu V, Lipsitch M (2012) Improving the Estimation of Influenza-Related Mortality Over a Seasonal Baseline. Epidemiology 23: 829-838.

2. Goldstein E, Cobey S, Takahashi S, Miller JC, Lipsitch M (2011) Predicting the epidemic sizes of influenza A/H1N1, A/H3N2, and B: a statistical method. PLoS Med 8: e1001051.

3. Baxter R (2010) Surveillance lessons from first-wave pandemic (H1N1) 2009, Northern California, USA. Emerg Infect Dis 16: 504-506.

4. Olson DR, Heffernan RT, Paladini M, Konty K, Weiss D, et al. (2007) Monitoring the impact of influenza by age: emergency department fever and respiratory complaint surveillance in New York City. PLoS Med 4: e247.

5. Smyth RL, Openshaw PJ (2006) Bronchiolitis. Lancet 368: 312-322.

6. Henrickson KJ, Hoover S, Kehl KS, Hua W (2004) National disease burden of respiratory viruses detected in children by polymerase chain reaction. Pediatr Infect Dis J 23: S11-18.

7. Christakis DA, Cowan CA, Garrison MM, Molteni R, Marcuse E, et al. (2005) Variation in inpatient diagnostic testing and management of bronchiolitis. Pediatrics 115: 878-884.

8. Swingler GH, Hussey GD, Zwarenstein M (1998) Randomised controlled trial of clinical outcome after chest radiograph in ambulatory acute lower-respiratory infection in children. Lancet 351: 404-408.
